# Implications of a fuel spill in the Bering and Chukchi Seas for two sentinel marine species: A simulation study

**DOI:** 10.1002/eap.70309

**Published:** 2026-09-20

**Authors:** Susannah P. Woodruff, Ryan R. Wilson, Irina S. Trukhanova, Deborah P. French‐McCay, Matthew Frediani

**Affiliations:** ^1^ Marine Mammals Management US Fish and Wildlife Service Anchorage Alaska USA; ^2^ North Pacific Wildlife Consulting, LLC Seattle Washington USA; ^3^ RPS Ocean Science, Tetra Tech Inc. South Kingstown Rhode Island USA; ^4^ Present address: Alaska Department of Fish and Game Division of Wildlife Conservation Douglas Alaska USA

**Keywords:** Bering Strait, Chukchi Sea, Northern Sea Route, oil spill, Pacific walrus (*Odobenus rosmarus*), polar bear (*Ursus maritimus*), Russia

## Abstract

Sea‐ice decline has opened Arctic shipping routes lengthening the maritime transit season. Along the Northern Sea Route (NSR), vessel traffic is projected to increase primarily from natural resource development‐related traffic. The NSR traverses the range of the Chukchi Sea (CS) polar bear (*Ursus maritimus*) subpopulation, and Pacific walrus (*Odobenus rosmarus divergens*) haulouts dot Russian islands and mainland, the Alaska coast, and the Bering Strait along the NSR. Oil spills are a primary threat to marine Arctic wildlife from maritime traffic with the potential to affect many individuals. We estimated the number of polar bears and walruses potentially exposed to oil from a CS or Bering Strait ship‐based spill. We simulated five large‐scale boat‐based spills in two NSR locations in August and October for crude and marine gas oil (MGO). We overlaid polar bear radio collar locations onto trajectories to estimate the number exposed. If oil was ≤0.5 nm from a haulout, we deemed the haulout exposed. We report the most impactful scenario to help managers and emergency responders understand the upper levels of impact to these species and inform response planning. Crude oil remained on the surface for the entire 60‐day simulations and >20% stranded onshore. Little surface MGO remained after 5 days or reached the shoreline. The Chukchi October crude spill exposed 24% of radio‐collared polar bears to lethal and sublethal oil, or 711 (95% CI = 438–1052) individuals as a proportion of the total population (*n* = 2937). During the Chukchi August crude spill, oil exposure occurred at ≥0.50 probability at four walrus haulouts. No polar bears or walrus haulouts were exposed to lethal levels of MGO from a spill. Our simulations suggest an oil spill would contaminate large areas of the marine environment and could have significant consequences for polar bears and walruses. The inability to conduct timely, effective cleanup underscores the importance of understanding and attempting to mitigate an Arctic oil spill. Spill preparation is expensive, time‐consuming, and increasingly challenging geopolitically, yet a lack of preparation could have catastrophic consequences to marine life, regional subsistence users, and the fishing and tourism industries.

## INTRODUCTION

Throughout the Arctic, climate warming continues to cause rapid thinning and retreat of sea ice (Kumar et al., 2020; Stroeve & Notz, 2018; Yadav et al., 2020). Sea ice loss in the Arctic has progressed faster than most climate models have predicted (Jahn et al., 2024; Stroeve & Notz, 2018), and the 18 lowest sea‐ice extents ever recorded have been in the last 18 years (NSIDC, 2024). This climate change‐induced sea‐ice decline has opened previously impassable (i.e., frozen) Arctic Ocean shipping routes (Smith & Stephenson, 2013), thus lengthening the historically short (i.e., July–Septemeber) Arctic maritime transit season (Boylan & Elsberry, 2019; Stephenson et al., 2011).

**FIGURE 1:**
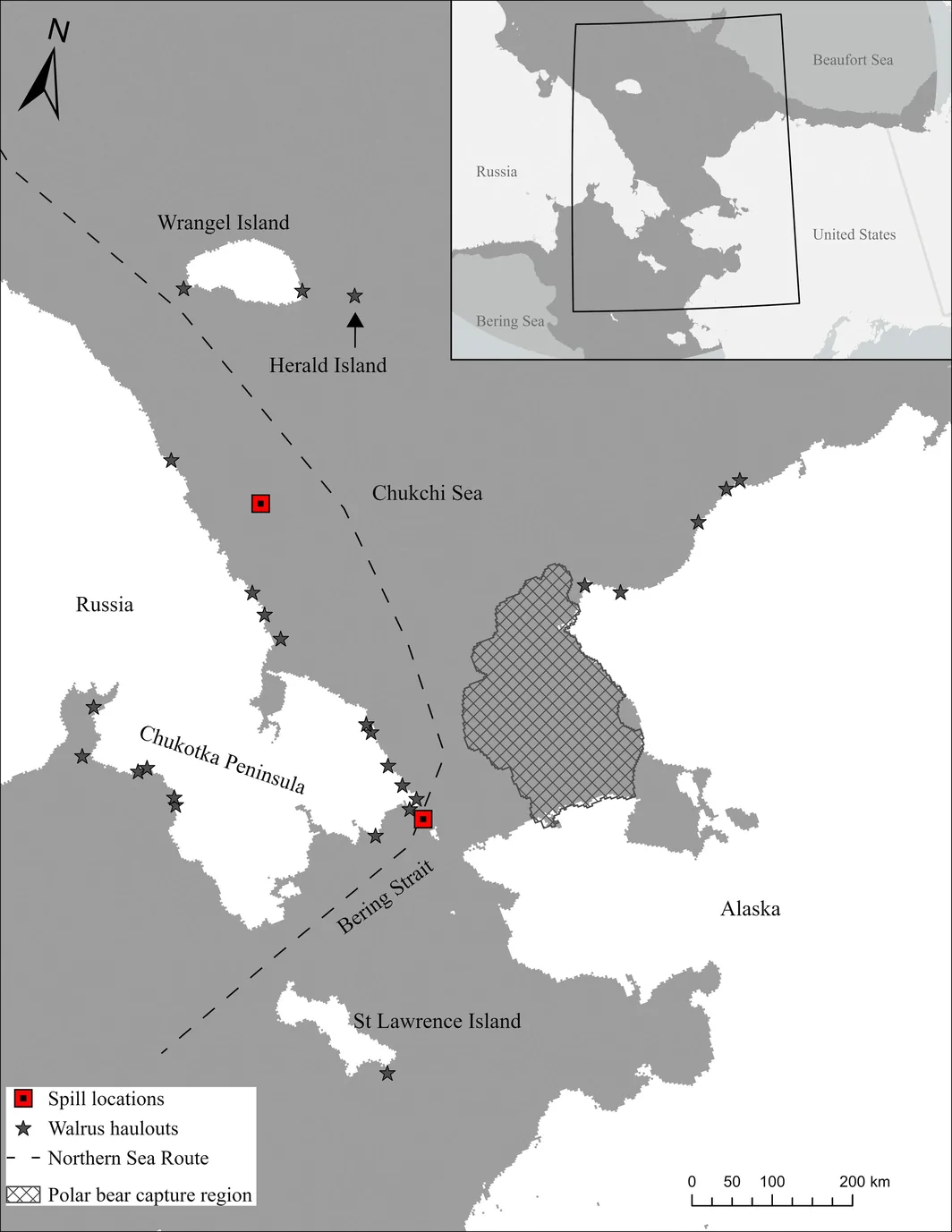
Study area for the analysis of the impact of simulated oil spills on polar bears and walruses in northern Alaska and Chukotka, Russia. Walrus haulouts (as identified by Fischbach et al., 2016) within our primary area of interest are denoted with stars. The hash marked area represents the region where polar bears were captured on the sea ice in the Chukchi Sea off the coast of Alaska between Kotzebue and Cape Lisburne in March and April from 2008 to 2017.

The primary marine transport route in the Arctic Ocean is the transoceanic Northern Sea Route (NSR). The NSR follows the western coast of Norway, northern and eastern coast of Russia, through the Arctic Ocean and the Bering Sea, and passes through the narrow Bering Strait, which separates Alaska and the Chukotka Peninsula, Russia (Figure 1). This region harbors one of the most productive marine ecosystems in the world with abundant fish, marine mammals, birds, and other sea life (Grebmeier et al., 2006; Hartsig et al., 2012; Sakshaug, 2004). The region is also important for providing invaluable subsistence resources to indigenous peoples as the cultural identity and food security of many coastal indigenous communities in both the United States and Russian Federation depend on a variety of marine mammals and fish species (e.g., Fall et al., 2013; Hill, 2017; Hovelsrud et al., 2008; Zdor, 2023). As summer sea ice extent is forecasted to continue to decline through the 21st century (Barnhart et al., 2016; Douglas & Atwood, 2022; Overland & Wang, 2013) annual vessel traffic is projected to increase 48%–67% (*n* = 124–171; USCMTS, 2019). Traffic related to natural resource development and activities, including oil and liquefied natural gas (LNG) shipments, is projected to make up the majority of the increase (USCMTS, 2019). In September 2023, as a result of European Union sanctions on importing Russian oil, Russia permitted the first shipment of crude oil in a non‐ice‐class oil tanker along the NSR from Russia to China through the Bering Strait (Humpert, 2023b) and plans to make this routine (Humpert, 2023a). A total of 10 oil tankers transited the NSR in 2023 (two in non‐ice‐class vessels; Wainwright, 2023). Increased transit by vessels carrying petroleum raises the risk of a spill (AMAP, 2010), and maritime travel in ice‐covered waters or by non‐ice‐class vessels further raises the risk and the resulting potential impacts (Bennett et al., 2020). Although ongoing improvements in geospatial infrastructure are underway, navigation along the NSR remains challenging due to limited satellite coverage and outdated nautical charts (Hill et al., 2015; Mahmoud et al., 2024), and recent foreign sanctions on international satellite images further hinder navigation capacity (Gunnarsson, 2024).

The primary threat to Arctic ecosystems from marine traffic is an oil spill (Arctic Council, 2009). For polar bears (*Ursus maritimus*) and walrus (*Odobenus rosmarus divergens*), oil spills have the potential to affect a large number of individuals (Amstrup et al., 2006; Garlich‐Miller et al., 2011; Wilson et al., 2018, 2024) and their habitat (MacCracken, 2012; Wilson et al., 2018). The effects on polar bears from contact and ingestion of oil from eating contaminated prey or grooming present serious health concerns including anorexia, tissue damage from uremia, dehydration, anemia, renal failure, or death (Øritsland et al., 1981). Direct oiling of their fur can cause thermoregulatory and metabolic stress (Hurst, 1982; Øritsland et al., 1981). Walruses suffer irritation of the eyes, skin, and respiratory tract from direct contact with oil and risk ingestion through consumption of contaminated prey; prolonged exposure may cause permanent damage or death (St. Aubin, 1990). Additionally, polar bears and walruses are important to indigenous communities as subsistence‐harvested species (Voorhees et al., 2014; Voorhees & Sparks, 2012). Oil spills not only potentially reduce the number of animals available to harvest, but contaminated animals are unsafe to eat (Dudarev et al., 2019; Wright et al., 2022). Spilled oil present in the spring during ice breakup or autumn during ice formation presents a greater risk than in ice‐free or ice‐covered seasons because of the difficulties associated with oil spill cleanup in mixed, broken ice (EPPR, 2017; Wilson et al., 2018). Given the extreme Arctic conditions and the likely lengthy response time to a spill, the likelihood of oil being effectively removed in ice‐covered Arctic waters is low (AMAP, 2010; Pavlov, 2020; Wilkinson et al., 2017).

**FIGURE 2:**
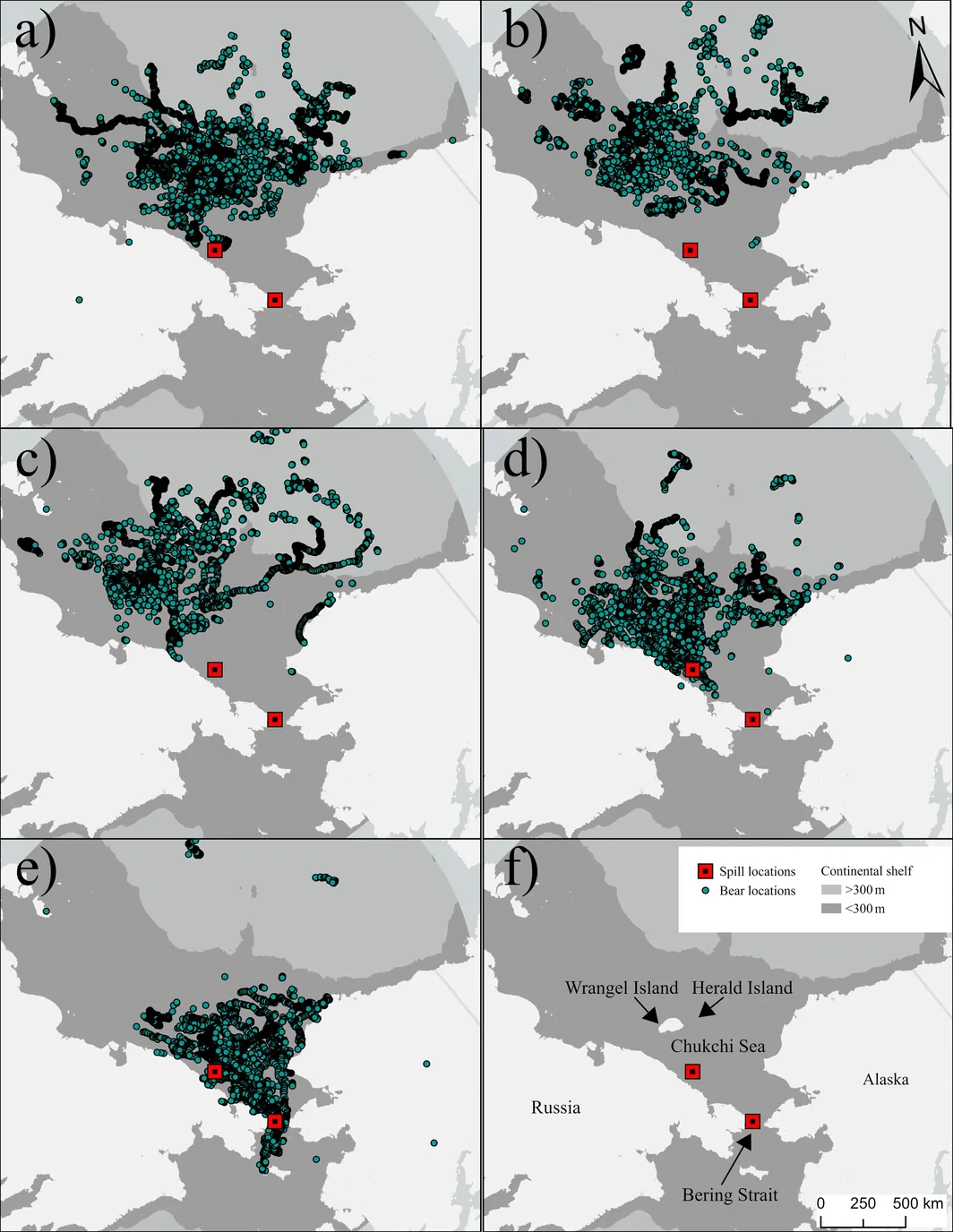
Locations of radio‐collared polar bears in the Chukchi Sea subpopulation from 2008 to 2017 in (a) August, (b) September, (c) October, (d) November, (e) December, (f) basemap and legend.

The NSR traverses the range of four of the 20 polar bear subpopulations including the Chukchi Sea (CS) subpopulation (Figure 1), which is shared between the United States and Russia. The boundaries of the CS polar bear subpopulation range extend from Alaska, USA, on the eastern boundary to Russia in the Eastern Siberian Sea to the west (Obbard et al., 2010), which includes Wrangel and Herald Islands and the Chukotka Peninsula, Russia. Wrangel Island and the Chukotka Peninsula provide important summer and autumn habitat where polar bears can wait for the re‐formation of sea ice while minimizing energy expenditure (Ware et al., 2017), and Wrangel provides the majority of maternal denning habitat (Rode et al., 2015). Based on satellite radio collar data collected from 1986 to 1994 (Garner et al., 1990) and 2008–2013 (Wilson et al., 2014, 2016), the movements and distribution of polar bears in the CS indicate a large proportion of bears spend substantial time every year during the ice‐free season on Wrangel and Herald Islands (Figure 2a–e; Rode et al., 2015) and the Chukotkan coast. Nearly all ships transiting the NSR pass between Wrangel Island and the Chukotkan coast (Kapsar et al., 2022, 2023).

Pacific walruses rest between bouts of foraging at sea‐ice and land‐based haulouts (Jay et al., 2011) in large groups (100 to >100,000 individuals) along the coast of Wrangel and Herald Islands and the northern coast of the Chukotka Peninsula from August through December (Robards & Garlich‐Miller, 2013). Walrus haulouts also occur along the CS coast of Alaska and in the Bering Strait region (Fischbach et al., 2016). Walruses forage on the shallow continental shelf, feeding mainly on benthic invertebrates such as bivalves and gastropods (Fay, 1982; Fischbach & Jay, 2016; Jay et al., 2011; Sheffield & Grebmeier, 2009), which provide the energy necessary to sustain their large body size and support reproduction. Sea ice decline has increased the use of coastal haulouts as walruses are forced to spend more time on land when sea ice platforms are unavailable (Fischbach et al., 2009; Kavry et al., 2008).

The objective of our study was to estimate the number of polar bears and walruses that could potentially come into contact with, and thus be contaminated by, oil from a ship‐based oil spill in the CS or Bering Strait. We focus on these regions because of the high biological and cultural importance (Grebmeier et al., 2006; Huntington et al., 2015) and the global concern for increasing vessel traffic leading to a catastrophic fuel spill in the region (AMAP, 2010; Arctic Council, 2009). First, we simulated five offshore oil spill scenarios along the NSR. We then overlaid polar bear location data from the CS polar bear subpopulation from 2008 to 2017 onto the simulated oil spill trajectories to evaluate the number of polar bears exposed to any sublethal and lethal levels of oil. To estimate potential impacts of an oil spill on walrus, we estimated the probability of walrus haulouts being oiled as a proxy to estimate the severity of effects to walruses impacted by the same five oil spill scenarios. Our goal was to report the worst‐case scenario results to help managers and emergency responders understand the potential upper levels of impact on these species and inform response planning.

## METHODS

### Polar bear locations and movement

We obtained location data for 110 radio‐collared adult female bears from a previous capture‐based study in the southern CS (Figure 1) and assessed which locations overlapped with the August–December period correlating with our simulated spills (Figure 2a–e). This resulted in a sample of 85 unique bears' movement, and 110 unique bear‐years. We considered unique bear‐years as independent samples. The 110 unique bear‐years consisted of 17 bears with >1 year of movement data and 68 bears with 1 year of movement data. Twelve bears had two unique years of data, two had 3 years of movement data, and three had 4 years of movement data. An average of 10 bears (SD = 6.5; range: 0–20) contributed movement data to each year of the analysis. The average number of locations per year (i.e., restricted to the period of simulated spills) across all animals was 6814 (SD = 5937; range: 225–19,157). On average, bears had 626 (SD = 624; range: 35–3236) locations that were used for this analysis.

Polar bear capture methods for these bears are described in Rode et al. (2015). Bears were immobilized and adult female bears were fitted with satellite radio collars (Telonics, Mesa, AZ, USA) collecting either Argos or global positioning system (GPS) locations at intervals ranging from every 2 h to every 4 days. Collars were programmed to automatically drop off the animals after being deployed for 1–2 years, thus females had the opportunity to utilize the entire range of the CS subpopulation, rather than simply the area where captures occurred. Adult male bears were not fitted with GPS collars because their heads are narrower than their necks meaning collars can be easily slipped off. Sub‐adult polar bears were also not fitted with GPS collars because they are still growing, and there are concerns their collars could become too tight before they were scheduled to drop off. Thus, location data was collected for only adult female polar bears.

To estimate polar bear locations at daily intervals, we used the continuous time correlated random walk (CRAWL; Johnson et al., 2008) model to account for differences in location acquisition rates and location error across collars. This also allowed us to match daily oil spill time‐steps to polar bear locations. To implement the CRAWL model, we used the “crawl” package (Johnson, 2022) in program R (R Core Team, 2024). We utilized error ellipse data to inform location uncertainty (McClintock et al., 2015) and obtained 25 posterior samples of possible trajectories based on the observed locations, time between locations, and their estimated error. Our approach was fully described in Wilson et al. (2018), and we provide our movement model code as Supplemental Information (i.e., “crawl_oilspill.R”). We used the same individual bears as Wilson et al. (2018) with an additional seven bears captured in 2017 included in this analysis. We estimated the number of bears that could come into contact with oil by overlaying polar bear locations with oil spill trajectories (described below). We provide code for this portion of the analysis as Supplemental Information.

### Walrus haulout locations and movement

For walrus, we chose to focus on large aggregations at haulouts as these are primary locations where the greatest number of walruses could be exposed to oil. Known walrus haulout locations (*n* = 34; Figure 1) were sourced from Fischbach et al. (2016). We selected coastal haulouts with documented regular use after the year 2000 and with ≥1000 individuals. Additionally, we considered only those haulouts where walruses were present between August and December. These criteria were chosen to focus the analysis on coastal haulouts that are both currently active and of sufficient size to represent sites where oil spills could affect a large number of walruses, ensuring that the simulated impacts are ecologically meaningful and relevant for conservation planning. We recognize oil spills occurring within concentrated, high‐benthic biomass foraging grounds (e.g., Hanna Shoal in the CS) could also pose a significant risk to large numbers of walruses. However, foraging areas are vast and walrus distribution can be sparse, with movement patterns largely impacted by ice conditions in a particular year (e.g., walruses either rest on ice nearby or go back to the haulouts). Limited walrus location and movement data during autumn (Jay et al., 2011) also prohibited us from conducting analysis similar to the polar bear analysis. Additionally, observing or mitigating the impacts of spills near foraging areas would be difficult due to the unpredictable and remote nature of these foraging locations. Furthermore, walruses are likely more vulnerable to spilled oil at high‐density haulouts where a singular access point makes contact with contaminated water nearly inevitable. In contrast, risk is more unpredictable at foraging areas as dispersed individuals are harder to track and may avoid the epicenter of an affected area.

Walruses are not present at all known haulouts every year, and their numbers can vary substantially from day to day (e.g., Altukhov et al., 2024; Fischbach et al., 2025), with individuals migrating between different parts of their range within a single season (Udevitz et al., 2017). However, for the purposes of this study, we assumed that all selected haulouts were at maximum occupancy throughout the study period to represent a worst‐case exposure scenario. The highest use haulouts include Cape Serdtse‐Kamen' (*n* ~ 184,000), Cape Vankarem (*n* ~ 47,000), and Kolyuchin Island (*n* ~ 42,855) in Russia and Point Lay Barrier Island (*n* ~ 78,000) in Alaska (Fischbach et al., 2016, 2025). Because occupancy varies from year to year, these numbers do not represent annual occupancy but instead represent the maximum haulout occupancy in a given year.

### Oil spill modeling

We simulated five oil spills that differed in their initial spill location, timing, and type of oil (Table 1). We simulated spills originating from two locations along the NSR: in the CS shipping lanes between the Bering Strait and Wrangel Island, Chukotka (68.826° N, 176.448° W; Table 1, Figure 1) and a southern location in the shipping lanes passing through the Bering Strait (65.931° N, 169.390° W; Table 1, Figure 1). The Chukchi spill location has experienced increased vessel traffic in recent years (Kapsar et al., 2022, 2023). The Bering Strait spill location is an area the U.S. Coast Guard has indicated as high concern for a spill (Knight, 2023). Both locations are within the range of CS polar bears and in the vicinity of multiple walrus haulouts (Figure 1).

**TABLE 1:** Model scenarios for simulated fuel spills in the Chukchi Sea and Bering Strait for Chayvo number 6 Russian crude oil (crude) and marine gas oil (MGO).

Simulation	Location	Latitude (°N)	Longitude (°W)	Oil type	Start month	Spill volume (bbl)
1	Chukchi Sea	68.826	176.448	Crude	August	720,000
2	Chukchi Sea	68.826	176.448	Crude	October	720,000
3	Chukchi Sea	68.826	176.448	MGO	August	20,000
4	Chukchi Sea	68.826	176.448	MGO	October	20,000
5	Bering Strait	65.931	169.390	Crude	October	720,000

*Note*: For all scenarios, release duration, simulation duration, and number of simulations were 3, 60, and 50 days, respectively. Locations are in World Geodetic System (WGS) 1984 projection.

We used two types of oil in the spill simulations: crude oil and marine gas oil (MGO; see Appendix S1: Table S1 for more on the oil types). For the crude oil spills, we assumed 720,000 bbl of crude oil would be released into the ocean to represent the full volume of oil transported by an Aframax tanker (assuming the tanker is 90% full; Papanikolaou et al., 2010), the primary tankers used to transport crude oil along the NSR (Melton, 2023). For the MGO spills, we assumed 20,000 bbl of MGO would be released into the ocean to represent an Aframax tanker losing its full fuel load (Papanikolaou et al., 2010). This loss of the full load of crude oil or MGO represents a worst‐case scenario oil spill resulting from, for example, an onboard explosion or fire, ship breakup in a severe storm, or a grounding event where both hulls are ruptured.

Finally, we simulated spills to begin during two different months in autumn: August and October. Minimum sea ice extent in the Arctic typically occurs in mid‐September. Sea ice subsequently begins reforming and begins to cover more southern reaches of the CS. Additionally, this is the period of the year when large aggregations of polar bears (e.g., 60 to ≥180 individuals on marine mammal carcasses; Laidre et al., 2018; Wilson et al., 2017) and walruses are on shore (Fischbach et al., 2016; Jay et al., 2017; Rode et al., 2015) or begin moving into the nearshore environment as the sea ice advances. For each of the five spill scenarios we considered, we modeled spills as releasing over a 3‐day period, with the oil released onto the ocean's surface, and then its fate was tracked for 60 days.

Coupled ice‐ocean models provide ocean currents, ice coverage, and ice drift velocities. We used inputs derived from the Copernicus Marine Global Ocean Reanalysis hydrodynamic model (GLORYS12V1; Jean‐Michel et al., 2021) provided by Copernicus Marine Service (CMEMS; https://doi.org/10.48670/moi-00021) to analyze water currents, ice movement, and ice coverage from 2016 to 2020. This model provides daily data at a 1/12° resolution across 50 vertical levels. This was integrated into the Spill Impact Model Application Package (SIMAP; French‐McCay, 2003, 2004; French‐McCay et al., 2018; French‐McCay, Jayko, et al., 2021) to model oil fate and transport. The SIMAP model quantifies the trajectory, areas swept by floating oil of varying thicknesses, and fates and concentrations of surface and subsurface oil components. The three‐dimensional physical fate model in SIMAP estimates distribution of whole oil and oil components on the water surface, on shorelines, in the water column, and on sediments. See Appendix S1: Section S1 for information on the environmental data we used to apply the oil spill model. This model has been used in multiple risk assessments for polar bears in the Arctic (e.g., Wilson et al., 2018, 2024) and validated with data from >20 large oil spills including the Exxon Valdez, North Cape, and Deepwater Horizon oil spills (e.g., French & Rines, 1997; French‐McCay, 2003, 2004; French‐McCay, Jayko, et al., 2021; French‐McCay, Robinson, et al., 2021), as well as with drifters (i.e., satellite‐tracked buoys) in Arctic ice (French‐McCay et al., 2017). These studies indicated oil trajectories depended primarily on wind, ocean current, and sea ice (when applicable) data inputs into the model.

We used stochastic modeling to evaluate the likelihood of a location being exposed to oil. The likelihood is based on statistical analysis of 50 individual trajectories of each spill scenario but with a randomly chosen start date within the month (August or October) of the scenario between 2016 and 2020 (the most recent 5‐year period where current and ice model data needed to run the oil spill model were available). Randomly drawing a start date across a 5‐year period allowed for each spill scenario to be analyzed under varied environmental conditions (e.g., wind direction and speed, currents, sea ice) that can be present in the Arctic Ocean. For each scenario we then aggregated the 50 spill iterations to estimate the overall probability of an area getting exposed to oil from an individual spill. To demonstrate the most adverse impact, we evaluated and report the most impactful case (MIC) from each set of stochastic runs for which the longest stretch of shoreline was oiled with >100 g/m^2^ for a scenario.

The potential for wildlife mortality is directly linked to the concentration of oil present on a given surface, typically measured as a mass per unit area in grams per square meter (g/m^2^). This metric serves as a critical indicator of both the oil's persistence in the environment and its capacity to cause physical harm (French‐McCay, 2016). On the water's surface, whereas concentrations between 1 and 10 g/m^2^ are associated with sublethal effects, acute lethal impacts are typically observed once concentrations exceed 10 g/m^2^ (French McCay et al., 2018; French‐McCay, 2016; Wilson et al., 2018, 2024). For oil that has washed ashore, the threshold for potential lethality increases significantly, with densities of ≥100 g/m^2^ required to cause fatal oiling of wildlife (French McCay et al., 2018; French‐McCay et al., 2011). Thus, our interest was not simply about the probability of wildlife interacting with any oil, but instead with densities of oil that have the potential to affect the survival of individuals that encounter it. This information was readily obtained from our spill simulation data.

### Impacts to polar bears

For each of the 50 iterations, for each of the five spill scenarios, we calculated how many polar bear movement trajectories (see *Polar bear locations and movement* above) came within 1 km of areas with ≥1 and ≥10 g/m^2^ of oil on the ocean's surface, ≥100 g/m^2^ on the shoreline, or passed through a location with any concentration of oil on the ocean surface or coastline. We assessed this for all 25 CRAWL realizations for each animal's trajectory to account for uncertainty in the movement paths for each bear. We assumed that radio‐collared adult females were a representative sample of space use and movement patterns for polar bears in the CS subpopulation given the similar resource selection patterns noted between adult females and adult males and sub‐adults (Wilson et al., 2022). While adult males and females show differing movement parameters (i.e., directional persistence and step length; Wilson et al., 2022), their similar resource selection estimates suggest they will occupy similar areas and thus face equivalent oil exposure risk. Differences in movement parameters between adult males and adult females in spring have further been attributed to males attempting to find mates (Laidre et al., 2013; Wilson et al., 2022), further indicating they likely occur in similar areas and thus experience similar levels of exposure to simulated oil spills.

Thus, we provide an estimate of the proportion of the subpopulation expected to be exposed to the different concentrations of oil across the different scenarios. This is the proportion of bears exposed to oil within 1 km at the end of the 60‐day spill tracking period (i.e., cumulative proportion). Additionally, we report the number of individuals this proportion represents in the total subpopulation based on the Regehr et al. (2018) CS abundance estimate (*n* = 2937 [95% Credible Interval = 1552–5944]) to help wildlife managers better understand the level of impact they may need to be prepared to deal with in the event of a spill. Finally, we report the number of days post‐spill when ≥1% of the sample population was exposed to oil at the same thresholds described above.

To account for variability in oil spill trajectories among the iterations of the simulation and animal movement trajectories among instances of the movement model and across individual tracked bears in our estimates of “impact”, we used the following bootstrapping procedure. Let $S=\left\{s_{i},\ldots ,s_{50}\right\}$ be the 50 unique spill simulations, $C_{i}=\left\{c_{i1},\ldots ,c_{i25}\right\}$ be the 25 CRAWL iterations of individual *i*, where i=1,…,110, and $I=\left\{1,\ldots ,110\right\}$ be the set of 110 individuals. Further, let B=1000 be the total number of bootstrap replicates. For each bootstrap (*b*) we drew a random sample of spill simulations $S^{\left(b\right)}=\left(s_{1}^{\left(b\right)},\ldots ,s_{110}^{\left(b\right)}\right)$ where $s_{j}^{\left(b\right)}$ is sampled from *S* with replacement (where *j* is one of the 110 samples retained for the bootstrapping). Next, for each bootstrap (*b*), we drew a sample of individuals $I^{\left(b\right)}=\left(i_{1}^{\left(b\right)},\ldots ,i_{110}^{\left(b\right)}\right)$ where $i_{j}^{\left(b\right)}$ is sampled from *I* with replacement. Lastly, for each sampled individual (*i*) in bootstrap *b*, we sampled a random CRAWL output $C^{\left(b\right)}=\left(c_{1}^{\left(b\right)},\ldots ,c_{110}^{\left(b\right)}\right)$ where $c_{j}^{\left(b\right)}$ is samples from $C_{i_{j}^{\left(b\right)}}$ with replacement. Thus, for each boostrap index *j*, $\left(i_{j}^{\left(b\right)},c_{j}^{\left(b\right)},s_{j}^{\left(b\right)}\right)$ defines one randomly paired individual path, CRAWL iteration, and spill simulation. For each bootstrap index *j*, we then determined if the individual came within 1 km of the simulated spill ($X_{j}^{\left(b\right)}$) such that $X_{j}^{\left(b\right)}=\left\{\begin{array}{cc} 1, & \;\text{if the path from}\;c_{j}^{\left(b\right)}\;\text{for individual}\;i_{j}^{\left(b\right)}\\ & \;\text{was}\leq 1\;\mathrm{km}\;\text{from spill simulation}\;s_{j}^{\left(b\right)},\\ 0, & \;\text{otherwise}. \end{array}\right.$

We then calculated the proportion (${\hat{p}}^{\left(b\right)}$) of individuals in bootstrap *b* that came into contact with oil:

$${\hat{p}}^{\left(b\right)}=\frac{1}{110}\sum_{j=1}^{110}X_{j}^{\left(b\right)}.$$

We also estimated the daily cumulative proportion of the population that was exposed to oil within 1 km using the same above bootstrapped dataset. The only difference was how *X* and *p* were calculated. To estimate the cumulative proportion of bears that were exposed to oil across days ($Y_{j,d}^{\left(b\right)}$), we calculated $Y_{j,d}^{\left(b\right)}=\left\{\begin{array}{cc} 1, & \;\text{if the path from}\;c_{j}^{\left(b\right)}\;\text{for individual}\;i_{j}^{\left(b\right)}\text{was}\leq 1\;\mathrm{km}\\ & \;\text{from spill simulation}\;s_{j}^{\left(b\right)}\mathrm{on}\;\text{or before}\;\mathrm{day}\;d\\ 0, & \text{otherwise} \end{array}\right..$

We then calculated the daily proportion (${\hat{p}}_{d}^{\left(b\right)}$) of individuals in bootstrap *b* that came into contact with oil on day *d* or earlier:

$${\hat{p}}_{d}^{\left(b\right)}=\frac{1}{110}\sum_{j=1}^{110}Y_{j,d}^{\left(b\right)}.$$

### Impacts to walrus

For each of the 50 iterations in each spill scenario, we considered a walrus haulout exposed if oil (i.e., for different concentrations mentioned above) came to within 0.5 nm of the haulout during the time when that haulout is traditionally used. This buffer distance is based on U.S. Fish & Wildlife Service guidelines that recommend medium‐size vessels maintain a 0.5 nm setback from walrus haulouts to avoid disturbance and minimize disruption of resting and behavior because walrus loiter in the water near haulouts (USFWS, 2025). Because walruses may move between haulouts in a given year, and all we have are the maximum number of walruses at a haulout (Table 2), we could not estimate the total number of walruses exposed given the potential for double‐counting of some walruses. For our purposes, we considered probability of exposure of ≥0.50 to be a threshold between oil being likely (i.e., ≥0.50) to occur at a site versus less likely (i.e., <0.50). We did not conduct a bootstrapping procedure for the walrus analysis, because unlike for the polar bear analysis, we did not need to account for uncertainty in the movement process. Thus, our assessment was simply the proportion of spill iterations that impacted walrus haulouts as described above.

**TABLE 2:** Walrus haulout in simulations used to estimate potential impacts of an oil spill on walrus in the Chukchi Sea and Bering Strait.

Haulout name	Location	Latitude	Longitude	Max. walrus	Months of use
Barrier Islands N of Pt Lay	Alaska	70.211	−162.281	1500	Aug–Oct
Cape Blossom	Wrangel Island, Russia	70.783	−178.769	76,702	Sept–Oct
Cape Inchoun	Chukotka Peninsula, Russia	66.248	−170.165	3252	Aug–Nov
Cape Inkigur	Chukotka Peninsula, Russia	66.735	−171.380	1686	Sep–Nov
Cape Kekurny	Chukotka Peninsula, Russia	66.136	−169.697	6500	Sep–Sep
Cape Lisburne	Alaska	68.883	−166.214	5000	Aug–Oct
Cape Newenham	Alaska	58.656	−162.124	5444	Jun–Dec
Cape Nunyamo	Chukotka Peninsula, Russia	65.627	−170.539	20,000	Sep–Nov
Cape Onmyn	Chukotka Peninsula, Russia	67.659	−175.268	9000	Aug–Oct
Cape Pe'ek	Chukotka Peninsula, Russia	66.007	−169.813	10,000	Sept–Nov
Cape Peirce	Alaska	58.570	−161.756	12,500	Aug–Dec
Cape Retkyn	Chukotka Peninsula, Russia	65.530	−177.172	2360	Jun–Sep
Cape Schmidt	Chukotka Peninsula, Russia	68.931	−179.482	50,000	Aug–Oct
Cape Seniavin	Alaska	56.399	−160.147	1000	Apr–Sep
Cape Serdtse‐Kamen'	Chukotka Peninsula, Russia	66.808	−171.569	184,000	Sep–Nov
Cape Unikyn	Chukotka Peninsula, Russia	66.421	−170.682	10,000	Oct–Nov
Cape Vankarem	Chukotka Peninsula, Russia	67.848	−175.814	47,000	Sep–Nov
Cape Waring	Wrangel Island, Chukotka	71.265	−177.514	2400	Jul–Sep
Corwin Bluff	Alaska	68.875	−165.102	1200	Aug–Sep
Erulya Spit	Chukotka Peninsula, Russia	66.030	−178.920	3000	Aug–Sep
Hagemeister Island	Alaska	58.580	−161.075	2941	May–Aug
Herald Island	Chukotka, Russia	71.413	−175.776	3500	Jul–Oct
Icy Cape	Alaska	70.327	−161.881	7500	Aug–Oct
Kaymatkyn Bay	Chukotka Peninsula, Russia	65.317	−175.921	3000	Aug
Kitovaya Spit	Chukotka Peninsula, Russia	65.603	−176.977	1421	Oct
Kolyuchin Island	Chukotka, Russia	67.456	−174.605	42,855	Aug–Oct
Me'eskyn Spit Island	Chukotka, Russia	65.472	−178.723	4700	Jul–Sep
Oksenof Point	Alaska	54.899	−164.358	2073	Sept–Nov
Pica Creek Mouth	Chukotka, Russia	62.521	178.118	7000	Jul–Sep
Point Lay Barrier Island	Alaska	69.795	−163.032	78,000	Aug–Oct
Punuk Islands	Alaska	63.080	−168.809	1000	Oct–Dec
Retkyn Spit	Chukotka Peninsula, Russia	65.390	−176.022	2360	Jun–Oct
Round Island	Alaska	58.604	−159.974	5000	Apr–Aug
Russkaya Koshka Spit	Chukotka Peninsula, Russia	64.571	178.592	4100	Jul–Aug

*Note*: Data represent the maximum number of walruses estimated to be at each haulout and the months when walruses are expected to be present.

## RESULTS

### Oil spill behavior—Chukchi Sea

#### Crude oil

**FIGURE 3:**
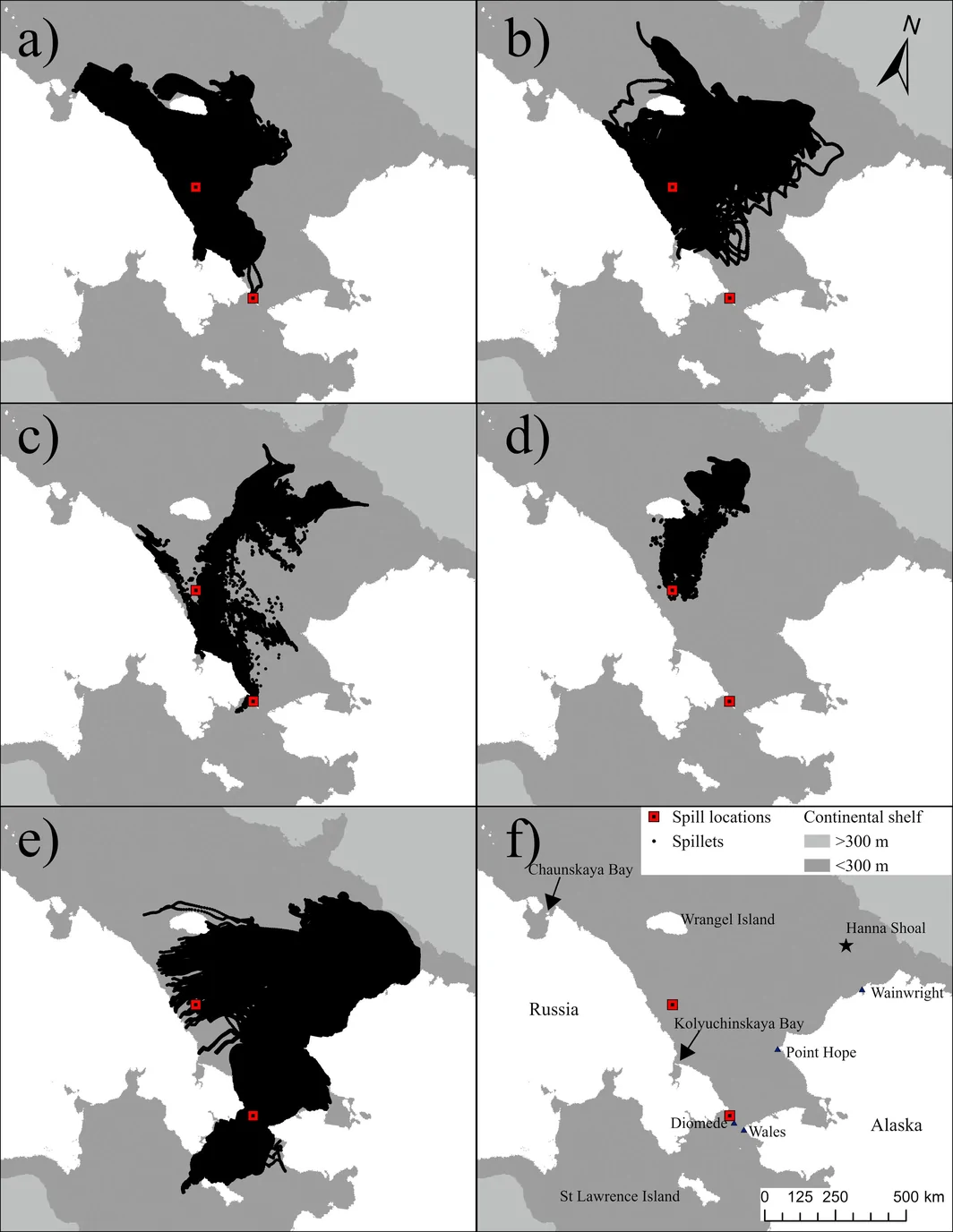
Example of surface oil spread over 60 days post‐release from a representative iteration of oil spills from each of the five scenarios analyzed in the study: (a) August crude oil release into the Chukchi Sea; (b) October crude oil release into the Chukchi Sea; (c) August marine gas oil release into the Chukchi Sea; (d) October marine gas oil release into the Chukchi Sea; (e) October crude oil release in the Bering Strait; (f) basemap and legend.

Our simulations indicated oil spread radially (Figure 3a–e). There were only slight differences in the distribution of oil 60 days after its initial release between the August and October release dates when comparing the same fuel type (Table 3). Areas with >80% probability of surface oil exceeding 1 g/m^2^ extended >75 km generally south and southwest from the release location in August and 200 km south and 80 km to the north in October (Appendix S1: Figure S1). Because the oil exposure probabilities were calculated from statistical analysis of the ensemble of individual trajectories for each release scenario, probability should be interpreted as: “The probability that any one specific area may experience contamination above the specified threshold is X%.” The probabilities do not imply the entire area would be covered with oil in the event of a spill, nor do they inform the quantity of oil in a given area. The surface area exposed to oil >1 g/m^2^ from a crude spill was 1–4 million km^2^. In August, surface oil >1 g/m^2^ would reach the Bering Strait in ~10 days and in 45–60 days to Chaunskaya Bay, the western edge of the spill. Surface oil >1 g/m^2^ took slightly longer (~20 days) to reach the Bering Strait in October but only 25 days to reach Chaunskaya Bay, again the western edge of the spill. Lethal levels of shoreline oil (100 g/m^2^) were reached with >90% probability 75–90 km southwest along the Chukotka coast 3–5 days after the release and reached the Alaska coastline 35–38 days post‐spill, albeit with a lower (1%–10%) probability of exceeding the lethal threshold. Depending on the environmental conditions and timing of the spill, an average of 700–790 km (range: 260–1460 km) of shoreline could be oiled.

**TABLE 3:** Stochastic results at 1 g/m^2^ threshold for the high probability surface oiling footprint (>80% probability).

Simulation no.	Direction	Distance	Time to shore	Time to AK coast	Length of coast oiled
1	South	200	5	38	700; 350–1460
2	South	200	3	35	790; 260–1240
3	All^a^	5	10^b^	NA	<107
4	All^a^	4	12^b^	NA	<164
5	North	270	2^c^	10^d^	930 (320–1560)

*Note*: Greatest distance from spill (distance; in kilometers), time to shore (in days; any shoreline), time to Alaska coast (AK; in days), and mean length and range (in kilometers) of coast oiled at a 100 g/m^2^ threshold. Simulations 1, 2, and 5 were crude oil; simulations 3 and 4 were marine gas oil.

^a^ No specific direction in radius around spill.

^b^ <10% probability of reaching the Chukotka coast.

^c^ Approximately 17 km southeast of the release location on the Diomede Islands. Higher probability shoreline oiling (>40%) extended to the northwest along the Chukotka coast to Neshkan.

^d^ There was a lower probability of shoreline oiling ~25% reaching Wales and other western Alaska communities and < 10% probability of reaching south to St. Matthew Island and north to Wainwright, Alaska.

In the MIC, high concentrations of surface oil (>100 g/m^2^) extended ~100 km northwest in the August spill simulation, with concentrations of 1–25 g/m^2^ surrounding Wrangel Island and extending south ~250 km to Kolyuchinskaya Bay on the Chukotka Peninsula. In the October spill, concentrations of >150 g/m^2^ traveled south ~60 km. Shoreline oiling >100 g/m^2^ was predicted along the Chukotka coast from the Bering Strait ~550 km northwest to Chaunskaya Bay and on the eastern coast of Wrangel Island. On Wrangel Island, crude oil exceeding a threshold of 100 g/m^2^ reached the shoreline with 2%–42% probability depending on location.

#### Marine gas oil

The surface oiling footprint with >50% probability at the 1 g/m^2^ threshold remained within 4–5 km of the release location for MGO in both time periods. The surface area exposed to oil from a MGO spill was 24,000–156,000 km^2^. After 14 days, oil no longer exceeded a 1 g/m^2^ threshold on the surface. A low likelihood (<10% probability) of shoreline oiling was predicted (along the Chukotka mainland) within 10 days of the release. An average of 21–24 km (range = 0–164 km) of shoreline could be oiled depending on the conditions and timing of the spill. In the MIC scenario, high concentrations of surface oil (>150 g/m^2^) remained in the immediate vicinity (October) or within ~20 km of the spill (August), and oil exceeded 100 g/m^2^ along the Chukotka coast.

### Oil spill behavior—Bering Strait

At the 1 g/m^2^ threshold, the crude oil release in the Bering Strait resulted in surface oil extending >270 km north of the release location into the CS with >80% probability (Table 3). Shoreline oiling was predicted within the first 2 days of the release ~17 km southeast of the release location on the Diomede Islands and could reach the Alaska coast near Point Hope in <10 days. In the MIC, high concentrations of surface oil (>150 g/m^2^) traveled mainly to the south through the Bering Strait ~100 km from the release location with shoreline oiling >100 g/m^2^ predicted from St. Lawrence Island, northward through the Bering Strait, extending to Wainwright, Alaska. Approximately 1–4 million km^2^ and 930 km (range = ~230–160) of ocean surface and shoreline, respectively, could be oiled.

### Crude versus MGO

The MICs highlight the differences between the two oil types modeled. The crude oil oscillated between the surface and water column for the majority of the 60‐day simulation, due to alternating periods of high winds that entrained oil and light winds that allowed oil to resurface. Crude oil remained on the surface for the entirety of the 60‐day simulations and resulted in 20%–25% of the release volume stranding on the shorelines. The MGO dispersed readily in the water column resulting in very little surface MGO remaining after the first 5 days. The high percentage of MGO entraining in the water column resulted in higher biodegradation when compared to the crude oil. By the end of the simulations, ~40%–50% of both types of oil had evaporated. The MGO, however, reached peak evaporation faster than the crude oil, and little of the MGO ever reached the shoreline.

### Impacts to polar bears

#### Crude oil

Crude oil on the ocean's surface led to a greater number of bears potentially exposed to oil compared to crude oil onshore or MGO onshore or on the ocean's surface. Impacts to polar bears were the most significant from the Chukchi October crude oil spill with a cumulative mean of 24% (95% CI = 15%–36%) of radio‐collared individuals exposed to lethal (≥10 g/m^2^) and 24% (95% CI = 15%–36%) exposed to sublethal (≥1 g/m^2^) levels of surface oil by the end of the 60‐day period that the simulated spill was monitored (Table 4; Figure 3b). As a proportion of the total population (*n* = 2937; Regehr et al., 2018), this equals 711 (95% CI = 435–1049) and 711 (95% CI = 438–1052) individuals, respectively. The Bering Strait spill was the second most consequential with means of 17% (95% CI = 7%–27%) of radio‐collared individuals exposed to lethal (≥10 g/m^2^) and 17% (95% CI = 7%–27%) exposed to sublethal (≥1 g/m^2^) levels of surface oil (Table 4; Figure 3e). Based on these proportions of the total population, this equals 488 (95% CI = 198–783) individuals in each case.

**TABLE 4:** Estimated number (mean and 95% CI) of polar bears potentially exposed to surface and/or shoreline (shore) oil at thresholds of ≥1, ≥10, and ≥ 100 g/m^2^ from oil spills simulated in the Chukchi Sea (simulations 1–4) and Bering Strait (simulation 5).

Sim no.	Location	Threshold	Mean	95% CI	*N*	95% CI
1	Surf	1	0.05	0.01–0.09	141	30–275
1	Surf	10	0.05	0.01–0.09	141	30–275
1	Shore	100	0	0–0	0	0–0
1	Shore	any	0.05	0.01–0.09	131	30–258
1	Surf+Shore	any	0.06	0.02–0.11	180	58–333
2	Surf	1	0.24	0.15–0.36	711	438–1052
2	Surf	10	0.24	0.15–0.36	711	435–1049
2	Shore	100	0.00	0.00–0.00	0	0–0
2	Shore	any	0.10	0.03–0.19	300	93–548
2	Surf+Shore	any	0.28	0.18–0.41	822	515–1191
3	Surf	1	0.01	0.00–0.02	18	0–66
3	Surf	10	0.00	0.00–0.00	0	0–0
3	Shore	100	0.00	0.00–0.00	0	0–0
3	Shore	any	0.00	0.00–0.01	3	0–34
3	Surf+Shore	any	0.01	0.00–0.03	21	0–78
4	Surf	1	0.06	0.01–0.12	173	40–361
4	Surf	10	0.00	0.00–0.00	0	0–0
4	Shore	100	0.00	0.00–0.00	0	0–0
4	Shore	any	0.01	0.00–0.04	26	0–107
4	Surf+Shore	any	0.07	0.02–0.14	198	45–401
5	Surf	1	0.17	0.07–0.27	488	198–783
5	Surf	10	0.17	0.07–0.27	488	198–783
5	Shore	100	0.00	0.00–0.00	0	0–0
5	Shore	any	0.05	0.00–0.11	155	0–321
5	Surf+Shore	any	0.20	0.10–0.30	573	289–872

*Note*: Simulations (sim no.) 1, 2, and 5 were crude oil; simulations 3 and 4 were marine gas oil. Mean represents the proportion of the bootstrapped polar bear sample population (i.e., radio‐collared individuals) that came within 1 km of oil. *N* is the number of individuals this proportion represents based on the Regehr et al. (2018) Chukchi Sea polar bear abundance estimate (*n* = 2937 [95% CI = 1552–5944]). Location represents the location of oil on the surface (Surf), the shoreline (Shore) or the surface and shore combined (Surf+Shore).

**FIGURE 4:**
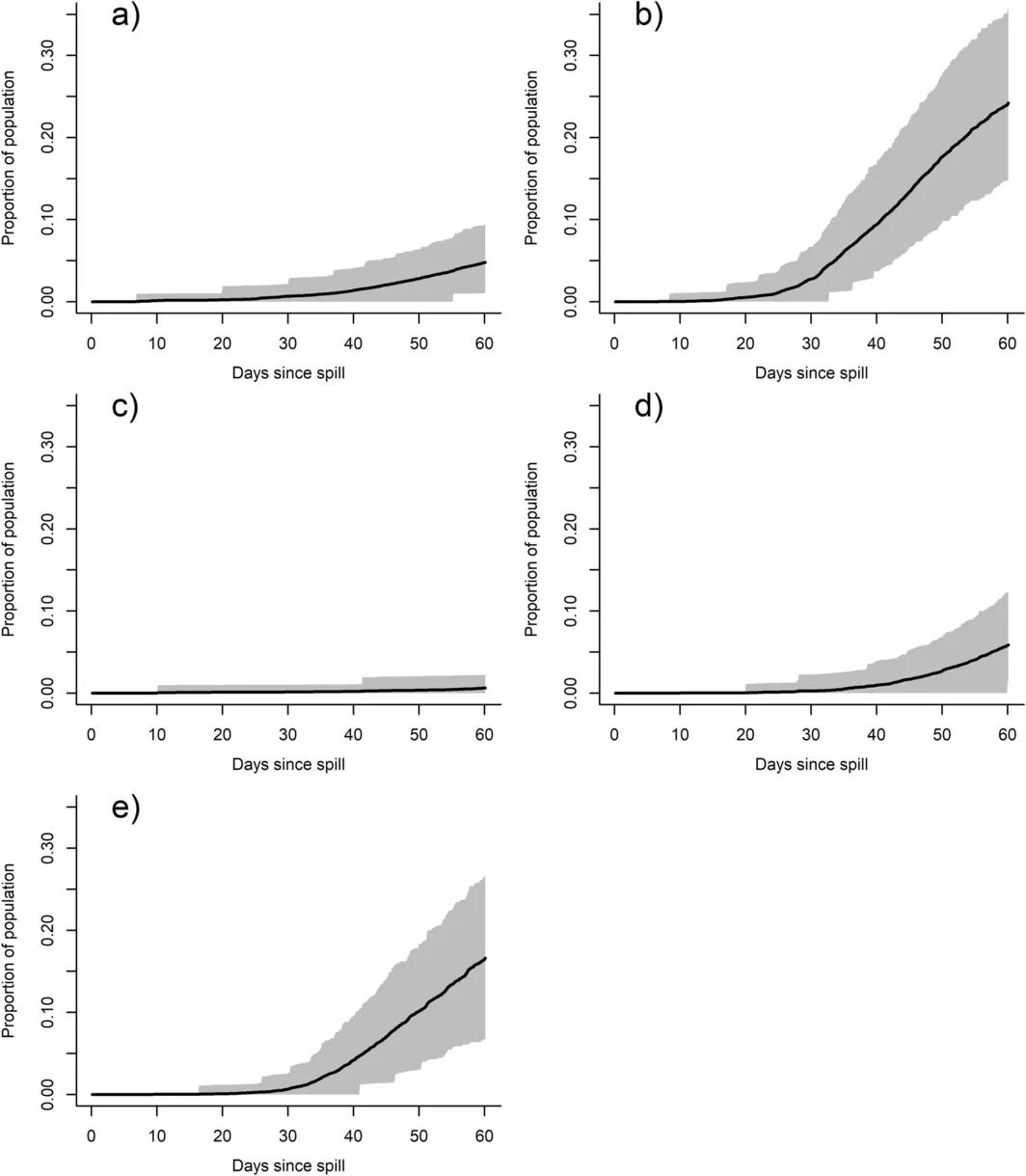
Cumulative proportion of bears exposed to oil (i.e., within 1 km) with concentrations ≥1 g/m^2^ over the 60 days the simulated spills were tracked. Results are for five different spill scenarios with different start dates, locations, and oil types. Solid lines represent the cumulative mean proportion of bears exposed to oil and the gray polygons represent the 95% CI for the estimated proportions. The scenarios represented in the figure are: (a) August crude oil release into the Chukchi Sea; (b) October crude oil release into the Chukchi Sea; (c) August marine gas oil release into the Chukchi Sea; (d) October marine gas oil release into the Chukchi Sea; (e) October crude oil release in the Bering Strait.

There were no crude oil simulations where bears were exposed to lethal levels of shoreline oil (≥100 g/m^2^; Table 4). The percent of radio‐collared bears that were exposed to any concentration of shoreline oil ranged from a low of <1% (95% CI = 0%–1%) for the August MGO spill to a high of 10% (95% CI = 3%–19%) for the Chukchi October crude spill (Figure 3c,d). These percentages correspond to 3 (95% CI = 0–34) to 300 (95% CI = 93–548) individuals as a proportion of the total population. The percentage of bears exposed to any level of surface or shoreline crude oil ranged from 6% (95% CI = 2%–11%; Chukchi August scenario) to 28% (95% CI = 18%–41%; Chukchi October scenario; Table 4; Figure 4). This represents 180 (95% CI = 58–333) and 822 (95% CI = 515–1191) individuals, respectively.

The number of days until ≥1% of the population was exposed to lethal and sublethal levels of surface oil was shortest for the Chukchi October crude spill (i.e., 25 days; Table 5). The time was slightly longer for the Bering Strait spill (i.e., 32 days), and the Chukchi August crude spill (i.e., 37 days). The number of days for shoreline oil of any threshold to expose ≥1% of the population was 29, 41, and 43 days for the Chukchi October, Bering Strait, and the Chukchi August crude spill, respectively. The time for any oil on the surface or shoreline (i.e., combined) was slightly shorter than for oil on the shoreline alone, at 24, 31, and 37 days for the Chukchi October, Bering Strait, and the Chukchi August crude spill, respectively.

**TABLE 5:** The number of days until ≥1% of the Chukchi Sea polar bear population was exposed to lethal and sublethal levels (surface) of oil or any oil threshold (shoreline) during oil spill simulations in the Chukchi Sea and Bering Strait.

	Location	Month	Oil threshold	No. days
Surface	Chukchi	Aug	≥1 g/m^2^	37
	Chukchi	Oct	≥1 g/m^2^	25
	Bering Strait	Oct	≥1 g/m^2^	32
Shoreline	Chukchi	Aug	>0 g/m^2^	37
	Chukchi	Oct	>0 g/m^2^	24
	Bering Strait	Oct	>0 g/m^2^	31

#### Marine gas oil

For both MGO spill simulations, no bears were exposed to lethal levels of oil on the surface or shoreline. For surface oil at the sublethal level, 0.6% (95% CI = 0%–2%) of radio‐collared bears were exposed during the August MGO spill and 6% (95% CI = 1%–12%) during the October MGO spill. This translates to 18 (95% CI = 0–66) and 173 (95% CI = 40–361) individuals in August and October, respectively. The proportion of radio‐collared bears exposed to any concentration of surface or shoreline MGO was 0.7% (95% CI = 0%–3%) and 7% (95% CI = 2%–14%), which translates to 21 (95% CI = 0–78) and 198 (95% CI = 45–401) individuals in August and October, respectively.

When considering exposure of ≥1% of the population, this occurred during only the October MGO spill to sublethal levels of surface MGO after 40 days and to any level of surface or shore MGO after 39 days.

### Impacts to walrus

**FIGURE 5:**
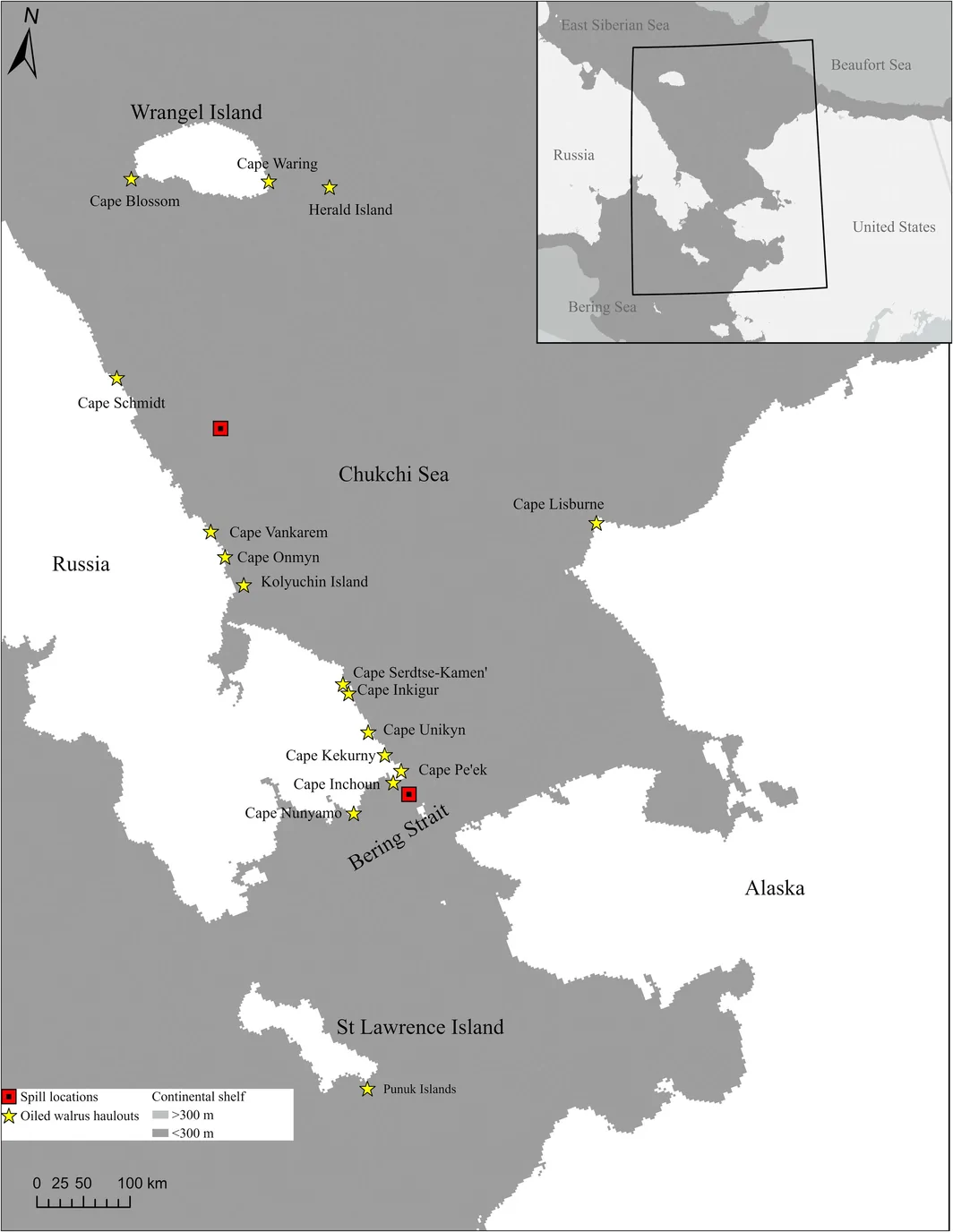
Map shows walrus haulout locations (Fischbach et al., 2016) exposed to any oil symbolized as yellow stars. Simulated oil spill locations are marked by red squares.

Across all spill scenarios, during the time walruses traditionally occupy the haulouts (Table 2), 47% (*n* = 16) of walrus haulouts were potentially exposed to oil and 53% (*n* = 18) were never exposed. Spills in August led to more walruses being exposed to oil than October spills. All potentially oiled haulouts except two (Punuk Islands and Cape Lisburne, Alaska) were in Russia (Table 2, Figure 5).

#### Crude oil

The Chukchi August crude spill was the most impactful for walruses. During this scenario, probabilities (*p*) were the same at all oiling levels (i.e., any oil ≥0.1 g/m^2^; sublethal ≥1 g/m^2^; lethal ≥10 g/m^2^), and there was a ≥ 0.50 probability of exposure to oil at four haulouts: Cape Schmidt (*p* = 0.51); Cape Vankarem (*p* = 0.62), Cape Onmyn (*p* = 0.83), and Kolyuchin Island (*p* = 0.85; Table 6). During the Chukchi October spill, only Cape Vankarem had a ≥ 0.50 probability (*p* = 0.85) of being exposed to lethal oil levels (Table 6) but in the Bering Strait October crude spill, five haulouts had ≥0.50 probability of being exposed to lethal oil levels: Cape Unikyn (*p* = 0.64), Cape Pe'ek (*p* = 0.57), Cape Inchoun (*p* = 0.64), Cape Inkigur (*p* = 0.60), and Cape Serdtse‐Kamen' (*p* = 0.56). The number of walruses oiled at the lethal level in each of these spills depends on haulout occupancy (see Table 2 for maximum number of walruses at each haulout).

**TABLE 6:** Probability of walrus haulout being exposed to oil at any (≥0.1 g/m^2^), sublethal (≥1 g/m^2^), or lethal (≥10 g/m^2^) levels.

Haulout	Scenario 1	Scenario 2	Scenario 3	Scenario 4	Scenario 5
Oil ≥0.1 g/m^2^					
Punuk Islands	0.000	0.000	0.000	0.000	0.015
Cape Lisburne	0.000	0.000	0.000	0.000	0.064
Cape Nunyamo	0.000	0.000	0.000	0.000	0.386
Cape Unikyn	0.041	0.061	0.068	0.000	**0.639**
Cape Pe'ek	0.058	0.000	0.068	0.000	**0.572**
Cape Kekurny	0.068	0.000	0.000	0.000	0.000
Cape Inchoun	0.133	0.040	0.151	0.000	**0.636**
Herald Island	0.145	0.018	0.000	0.000	0.000
Cape Waring	0.198	0.000	0.000	0.000	0.000
Cape Blossom	0.214	0.036	0.000	0.000	0.000
Cape Serdtse‐Kamen'	0.356	0.213	0.313	0.000	**0.561**
Cape Inkigur	0.401	0.189	0.265	0.000	**0.598**
Cape Schmidt	**0.508**	0.080	0.024	0.000	0.000
Cape Vankarem	**0.623**	**0.848**	**0.573**	0.234	0.400
Cape Onmyn	**0.828**	0.222	**0.644**	0.092	0.056
Kolyuchin Island	**0.850**	0.264	**0.757**	0.139	0.056
Oil ≥1 g/m^2^					
Punuk Islands	0.000	0.000	0.000	0.000	0.015
Cape Lisburne	0.000	0.000	0.000	0.000	0.064
Cape Nunyamo	0.000	0.000	0.000	0.000	0.386
Cape Unikyn	0.041	0.061	0.068	0.000	**0.639**
Cape Pe'ek	0.058	0.000	0.068	0.000	**0.572**
Cape Kekurny	0.068	0.000	0.000	0.000	0.000
Cape Inchoun	0.133	0.040	0.151	0.000	**0.636**
Herald Island	0.145	0.018	0.000	0.000	0.000
Cape Blossom	0.214	0.036	0.000	0.000	0.000
Cape Serdtse‐Kamen'	0.356	0.213	0.313	0.000	**0.561**
Cape Waring	0.198	0.000	0.000	0.000	0.000
Cape Inkigur	0.401	0.189	0.265	0.000	**0.598**
Cape Schmidt	**0.508**	0.080	0.024	0.000	0.000
Cape Vankarem	**0.623**	**0.848**	**0.573**	0.234	0.400
Cape Onmyn	**0.828**	0.222	**0.644**	0.092	0.056
Kolyuchin Island	**0.850**	0.264	**0.757**	0.139	0.056
Oil ≥10 g/m^2^					
Punuk Islands	0.000	0.000	0.000	0.000	0.015
Cape Lisburne	0.000	0.000	0.000	0.000	0.064
Cape Nunyamo	0.000	0.000	0.000	0.000	0.386
Cape Unikyn	0.041	0.061	0.000	0.000	**0.639**
Cape Pe'ek	0.058	0.000	0.000	0.000	**0.572**
Cape Kekurny	0.068	0.000	0.000	0.000	0.000
Cape Inchoun	0.133	0.040	0.000	0.000	**0.636**
Herald Island	0.145	0.018	0.000	0.000	0.000
Cape Blossom	0.214	0.036	0.000	0.000	0.000
Cape Serdtse‐Kamen'	0.356	0.213	0.000	0.000	**0.561**
Cape Waring	0.198	0.000	0.000	0.000	0.000
Cape Inkigur	0.401	0.189	0.000	0.000	**0.598**
Cape Schmidt	**0.508**	0.080	0.000	0.000	0.000
Cape Vankarem	**0.623**	**0.848**	0.000	0.000	0.400
Cape Onmyn	**0.828**	0.222	0.000	0.000	0.056
Kolyuchin Island	**0.850**	0.264	0.000	0.000	0.056

*Note*: Probabilities ≥0.50 are shown in bold. All haulouts potentially exposed to oil are in Russia, except Punuk Islands and Cape Lisburne, Alaska.

#### Marine gas oil

Similar to polar bears, for both MGO spill simulations, no walrus haulouts were exposed to lethal levels of oil. A ≥0.50 probability of sublethal or any level of surface oil occurred in only the MGO August spill at Cape Vankarem (*p* = 0.57), Cape Onmyn (*p* = 0.64), and Kolyuchin Island (*p* = 0.76).

## DISCUSSION

Our research couples simulated oil spill trajectories and animal location data to evaluate potential impacts of a spill on two species of protected marine mammals that are important subsistence resources for indigenous communities. Our simulations highlight the rapid spreading of oil in the marine environment, suggesting an oil spill would contaminate large areas and have significant consequences for polar bears and walruses. In the event of a crude oil spill in autumn, ~140 to >700 polar bears could potentially be exposed to surface oil, and both surface and shoreline oil could affect >100,000 walruses at haulouts and those transiting to and from haulouts during bouts of foraging. Acute mortality or chronic effects of exposure to any oil, such as lung disease, adrenal dysfunction, and suppressed immunity, significantly impair the reproductive success and survival of *K*‐selected marine mammals, such as polar bears and walruses, and could have long‐lasting population‐level impacts. Because *K*‐selected species are characterized by a relatively long lifespan, late maturity, small litter size, and multiple years of parental investment, these persistent sublethal injuries can prevent population recovery for decades (Ackleh et al., 2017; Barron et al., 2020; Ruberg et al., 2021).

Due to differences in the physical and chemical properties of oil types, the rate and mechanisms of oil weathering and degradation differ (Daling & Strøm, 1999; Wang et al., 1998). Although crude oil is light, when emulsified, it could persist much longer than MGO on the water surface (Lee et al., 2022; Tarr et al., 2016). Additionally, if ice were present during and/or after a spill, both crude and MGO could be trapped under ice and remain in the marine environment for a longer period of time (National Research Council et al., 2003; Wilkinson et al., 2017), though potentially less oil would come ashore while ice is present. However, when the ice eventually melts, the trapped oil could come ashore, possibly far from the spill site, impacting sites we did not consider in our analyses. The MIC, that is, the scenario with the longest stretch of shoreline oiled by >100 g/m^2^ of oil, occurred in conditions when ice was not present near the spill site, such that oil spread on the surface and moved the furthest, mostly transported by winds to the coast.

In general, crude oil spills were much more consequential than MGO spills, both in terms of surface area and shoreline oiled, and impacts to polar bears and walruses. The Chukchi crude oil releases largely impacted the Chukotka coast and Wrangel Island, whereas the smaller volume MGO releases primarily impacted a small area of the Chukotka coast. The Bering Strait crude oil release resulted in surface oil spreading north into the CS, oiling the coast of Alaska, and traveling south past St. Lawrence Island. Crude spills resulted in lethal levels of surface oil for both polar bears and walruses, whereas none of the simulated MGO spills resulted in lethal impacts for either species. This does not mean, however, there would be no impacts as direct and indirect exposure to even low levels of oil can be impactful for many years after a spill (Bergeon Burns et al., 2014; Peterson et al., 2003; Sullivan et al., 2019). Thus, we may have underestimated the number of individuals impacted given several of our assumptions.

We did not account for indirect effects of oil contamination, which include consumption of oiled prey (Helm et al., 2015; O'Hara & Becker, 2002), loss of habitat (Bergeon Burns et al., 2014; Sullivan et al., 2019), decreased reproduction and body condition (Sullivan et al., 2019; Takeshita et al., 2017, 2021), and decline in prey availability (Helm et al., 2015; O'Hara & Becker, 2002; Sullivan et al., 2019). Polar bears do not attempt to avoid oil (Derocher & Stirling, 1991) or consumption of oiled prey (Derocher & Stirling, 1991; Neff, 1990; St. Aubin, 1990), and we are not aware of avoidance being documented for walrus. As benthic foragers, walruses may be subject to the longer term indirect effects associated with consumption of oiled prey (Helm et al., 2015; Saadoun, 2015) and decline in prey availability (BOEM, 2015). For instance, Hanna Shoal, a major walrus foraging ground in one of the most biologically productive areas in the CS (Jay et al., 2012), is likely affected by our simulated oil spills, which could have long‐term effects on their foraging base. Additionally, we did not consider potential contamination of walruses hauled out on sea ice, as the sea ice conditions are variable year to year and distribution of walrus aggregations on ice is hard to predict. We also assumed there was no large‐scale attempt to decontaminate oiled individuals, as any decontamination and housing of such a large number of oiled animals would not be feasible given the risks to both animals and people associated with capture and transport and the specialized equipment and facilities needed for decontamination (Wilson et al., 2024). The nearest facility capable of decontaminating large marine mammals in the United States is in Prudhoe Bay, Alaska, >1000 km from either spill site, although the capacity is minimal at only two to three individuals. We are unaware of what equipment and capacity to decontaminate oiled wildlife exists in Russia.

Alternatively, we may have overestimated impacts given we assumed all polar bears within 1 km of oil and all walruses at a haulout within 0.5 nm of oil were potentially exposed to oil. This may not always be the case. For polar bears, we reported the estimated number of individuals potentially exposed to oil, and our results were similar to other simulations modeling the effects on polar bears of an underwater pipeline leak during autumn (October) at two planned areas of development in the CS (French‐McCay, 2016; French‐McCay et al., 2017; Wilson et al., 2018). They reported 100–800 polar bears in the CS subpopulation could be oiled (based on an estimated total of 2000 bears in the subpopulation; Wilson et al., 2018). Several other studies looking at the impact on polar bears from several simulated oil spills (sub‐sea pipeline leaks) in the southern Beaufort Sea suggested fewer individual bears (0–74 [Amstrup et al., 2006]; 3–38, [Wilson et al., 2024]) would be exposed to oil depending on the time of year and presence/absence of sea ice. For walrus, all we had is maximum haulout numbers, and occupancy of haulouts and number of individual walruses at a haulout can vary within and between years (Fischbach et al., 2016). Unlike for polar bears where we kept track of individual bears in our analysis, this was not possible with the walrus data, which could inflate the estimates of oil‐exposed walruses because there may be double‐counting. Nevertheless, the estimated probabilities of oiling highlight the potential for large numbers of walruses to be impacted in the event of an oil spill. Given the uncertainty in absolute counts, we urge the reader to focus on the relative probabilities of impact rather than the potential number of impacted walruses. These probabilities provide a more robust indication of risk and the potential scale of impact.

The majority of the spilled oil in our simulations remained within the range of the CS polar bear subpopulation, yet in some spill iterations, surface and shore oil spread into the ranges of both the Southern Beaufort Sea and Arctic Basin subpopulations. Additionally, there may be bears impacted that we are not accounting for in our analyses, such as those in the Laptev Sea subpopulation. This is particularly likely from oil on and around Wrangel Island as it is likely that some bears from this subpopulation visit the island during the ice‐free season (August–October).

Several other analyses have assessed the probability and scale of impacts from an Arctic oil spill (Helle et al., 2020; Pavlov et al., 2022) and the impacts to marine life (e.g., Helle et al., 2020; Nevalainen et al., 2018, 2019; Wilson et al., 2018, 2024). The probability of an area being oiled and the size of the impacted area vary due to many factors including location of spill, season, presence of sea ice, and weather (Helle et al., 2020; Pavlov et al., 2022). We chose our locations based on vessel tracking data (Kapsar et al., 2022) and U.S. Coast Guard areas of concern (Knight, 2023). However, our simulations assume specific, fixed scenarios that do not fully encompass the spatial and temporal variability of Arctic conditions. We acknowledge the limitations of our analysis given the point locations we chose for an oil spill to occur and the length of the NSR and the time of year of our simulations (i.e., August and October). By focusing on August and October, we potentially miss the worst‐case spill trajectories and weathering behavior that occur in high summer open water or under complete winter ice cover (ITOPF, 2025). Furthermore, our analysis focused on crude oil and MGO and did not account for the potential for, for example, spills of Very Low Sulfur Fuel Oil (VLSFO) and Ultra Low Sulfur Fuel Oil (ULSFO), which behave differently (i.e., they clump) than traditional crude oil, often resulting in lower cleanup efficacy in cold water environments (PAME‐EPPR, 2024).

This region of the CS serves as a critical ecological sanctuary in the Arctic for both polar bears and walruses. Wrangel Island provides important seasonal resting habitat for polar bears (Garner et al., 1994; Rode et al., 2015), and the majority (84%) of pregnant females in the CS subpopulation den on land (Rode et al., 2015), primarily on Wrangel and Herald Islands and the northeastern coast of the Chukotka Peninsula (Obbard et al., 2010; Ovsyanikov, 2005; Rode et al., 2015; Stishov, 1991). Most of the Pacific walrus population spends late summer and fall in the CS, within either Russian or US waters. In the absence of sea ice, coastal haulouts provide essential resting platforms between foraging trips and serve as refugia during storms and protection from predators. As Arctic sea ice continues to decline, the importance of coastal haulouts has increased, with only a few key sites (e.g., Point Lay, Cape Serdtse‐Kamen' or Cape Vankarem) hosting the majority of the population (Altukhov et al., 2024; Fischbach et al., 2022, 2025). Some of these major haulouts have been shown to be at risk in the event of an oil spill, with model simulations suggesting that up to 71% of the Pacific walrus population could be exposed to lethal concentrations of oil under certain spill scenarios (Beatty et al., 2022). Only two of the major haulouts in Alaska, Punuk Island, and Cape Lisburne were exposed to the simulated spills, both of which were exposed to oil from the October Bering Strait crude oil spill scenario.

The timing of start dates for the simulations coincides with the ice‐free season and the months of the highest vessel use along the NSR (i.e., August–November; Gunnarsson & Moe, 2021). We also chose to report the most impactful scenario to help managers and emergency responders understand the upper levels of impact on polar bears and walruses to inform spill response planning. Many factors can play into the level of exposure, including smaller spills, favorable weather, or spills closer to response resources, which may be less impactful to marine wildlife.

The Arctic is one of the most remote and difficult places to access on the planet. There has never been a large oil spill in Arctic waters, but the difficulty of cleanup from other large non‐Arctic spills (e.g., Exxon Valdez, 1989, Prince William Sound, Alaska, USA; Deepwater Horizon, 2010, Gulf of Mexico, USA) indicates cleanup in Arctic waters would be challenging (ITOPF, 2025; Ivshina et al., 2015; Pavlov, 2020). Technological limitations and harsh conditions make cleanup in a mix of oil and water extremely difficult. Standard cleanup methods (e.g., booms and skimmers or dispersants) designed for open‐water spills are often ineffective (ITOPF, 2025; Wilkinson et al., 2017). Even small amounts of ice can reduce the effectiveness of booms and skimmers, although ice can also act as a natural boom for the oil keeping it contained in calm waters (Wilkinson et al., 2017). Notwithstanding the concern for toxicity due to dispersant exposure (Wilkinson et al., 2017), dispersants may be effective in Arctic waters, although colder temperatures reduce their effectiveness (Lessard & DeMarco, 2000; Lewis & Daling, 2007).

Long‐term damage to the ecosystems in both of the aforementioned spills (e.g., Barron et al., 2020; Helm et al., 2015) also provide clues to the ecosystem impacts that could occur in a large‐scale Arctic oil spill. In our study, we assumed there was no cleanup effort for the duration of the simulations. This is a realistic assumption given the remote nature of the spill, inadequate resources, and Arctic weather and sea conditions limit the capacity for response to an oil spill (AMAP, 2010; Bi et al., 2025; National Research Council, 2014). We also assumed any spill was a complete loss as opposed to a partial spill. We recognize most oil tankers transiting the NSR will be double‐hulled following regulations (IMO, 2017), thus reducing the chances of a complete loss. However, since Russia's invasion of Ukraine, indications suggest an increase in a “shadow fleet” of vessels traversing the NSR, many of which are aging ships (i.e., >15 years old), lack ice‐class certification (permitted only July–15 November), and often sail with transponders turned off to avoid being tracked (Vakhrusheva et al., 2025). Additionally, extreme structural damage or uncontrolled fires can overcome cargo containment systems leading to the loss of the entire cargo (ITOPF, 2025). Given the extended response time (i.e., several days to weeks) and limited response resources (Vakhrusheva et al., 2025), a complete loss is not inconceivable.

A major factor in limiting damage from a spill is the time it takes to initiate cleanup (AMAP, 2010). Our simulations exhibit how quickly oil spreads from the spill site, and by the time resources arrive, the size of the oiled area could be substantial. For example, oil reached the shoreline 75 km southwest of the spill site within 3 days for the Chukchi October crude spill. The closest spill cleanup resources are located >150 km from this region of the NSR (Nome, Alaska, USA; Provideniya and Pevek, Russia) and thus we can expect extended response time. The response capabilities in these small communities are limited and operate only when the conditions are relatively ice‐free and waters are navigable. Moreover, historically the United States and Russia have cooperated on oil spill response coordination, though current geopolitical tensions have paused joint disaster preparedness response exercises (Fouche & Dickie, 2024), which could further hinder a more rapid response to a spill.

The primary objective of our study was to estimate the number of polar bears and walruses that could potentially be exposed to oil in the event of a ship‐based spill in the CS or Bering Strait. Model‐based frameworks for estimating oil spill trajectories and their impacts (e.g., this study, French‐McCay et al., 2017; Helle et al., 2020; Wilson et al., 2018, 2024) heighten our ability to predict outcomes and can be used to inform the response and cleanup capacity that would be needed to mount an effective response. Consistent with other studies, our research illustrates broad geographical reach and large‐scale impacts on the ecosystem and marine life within the footprint of an oil spill in our two spill locations, and likely most places in the Arctic. The inability to conduct timely, effective cleanup leading to oiling of Pacific walruses and polar bears and the cascading regional socioeconomic effects underscore the importance of understanding and attempting to mitigate the risks associated with an Arctic oil spill. For example, limiting ship transits to summer and early autumn (i.e., July–October) when sea ice is at its lowest, particularly for non‐ice‐class ships. Improved communication and navigational support (Hill et al., 2015) and stricter enforcement of regulations and agreements including the Polar Code (IMO, 2015), and increasing spill response capabilities in established ports, such as Pevek, Egvekinot, Anadyr, and Petropavlovsk‐Kamchatsky (in Russia), would be prudent. The recent addition of two emergency response tugs to the port of Petropavlovsk‐Kamchatsky increases response capabilities but capacity remains inadequate (Vakhrusheva et al., 2025). We also critically need to increase the response capacity for species such as polar bears and walruses, given, at this point, we have the capacity to handle only three polar bears, and that facility in Alaska is >1000 km away from anywhere on the NSR. A large spill would require a much larger network of response capability than exists today. Preparation for the impact of a spill is expensive, time‐consuming, and increasingly challenging given the current geopolitical circumstances, yet a lack of preparation could have catastrophic consequences not only to marine life but also to regional subsistence users and the fishing and tourism industries (Bi et al., 2025).

## CONFLICT OF INTEREST STATEMENT

The authors declare no conflicts of interest.

## Supporting information


Appendix S1:

## Data Availability

Data and novel code (Woodruff et al., 2026) are available in Dryad at https://doi.org/10.5061/dryad.3n5tb2rz6.
