# Supplementary material for: Implications of a fuel spill in the Bering and Chukchi Seas for two sentinel marine species: A simulation study
Source: Ecol Appl. 2026 Sep 20;36(6):e70309. doi: 10.1002/eap.70309 (PMC13589909; doi:10.1002/eap.70309)
Supplement: Supplementary file 1 [file EAP-36-e70309-s001.pdf]

**Appendix S1**

# Implications of a fuel spill in the Bering and Chukchi Seas for two sentinel marine species: A simulation study

Susannah P. Woodruff, Ryan R. Wilson, Irina S. Trukhanova, Deborah P. French-McCay, Matthew Frediani

*Ecological Applications*

**Section S1**

Environmental data used to develop the oil spill model

To implement the Spill Impact Model Application Package (SIMAP) model, we gathered data on coastline, bathymetry, and shore types and subsequent oil retention. Retention of oil on a shoreline depends on the shoreline type, width and angle of the shoreline, viscosity of the oil, the tidal amplitude, and the wave energy.

We obtained bathymetric data for the study area from the General Bathymetric Chart of the Oceans (GEBCO) Digital Atlas (2023), which consists of a global one arc-minute grid largely generated by combining quality-controlled ship depth soundings with interpolation between points guided by satellite-derived gravity data. We extracted a subset of the gridded GEBCO data to generate the depth grid used for an input to the SIMAP model.

We obtained data on ocean currents and ice coverage from the Copernicus Marine Global Ocean Reanalysis (E.U. Copernicus Marine Service Information; https://doi.org/10.48670/moi-00021). We acquired daily mean 3-dimensional current speed and direction, surface sea ice drift speed and direction, ice thickness, and ice coverage fraction for the time period of 2016–2020 as input to the SIMAP model.

We obtained wind data from the European Centre for Medium-Range Weather Forecasts (ECMWF) Reanalysis Product for the same five-year period, 2016–2020. The gridded outputs from ECMWF describe atmospheric as well as ocean-wave and land-surface conditions (Dee et al. 2011).

We obtained water temperature and salinity from the World Ocean Atlas 2018 (WOA18, Garcia et al. 2019), compiled and maintained by the National Centers for Environmental Information (NCEI) through US National Oceanographic Data Center (NOAA) (https://www.ncei.noaa.gov). Average surface water values were used in this analysis. The air immediately above the water is assumed to have the same temperature as the water surface, this being the best estimate of air temperature in contact with floating oil.

Suspended sediment concentration and sedimentation rate are assumed to be 10 mg/L and 0.2 cm/yr, respectively, typical values for shelf waters (Kullenberg 1982). This infers a settling rate of 1 m/day. The horizontal diffusion (randomized mixing) coefficient is assumed to be 1 m2/s and the vertical diffusion (randomized mixing) coefficient is assumed 1 cm2/s. These are reasonable values based on empirical data (Okubo and Ozmidov 1970; Okubo 1971) and modeling experience.

Because primarily Russian tankers transit this area, we selected parameters to reflect the types of oil and tankers used by the Russians. Oil from the Russian Chavyo oil field is well characterized (ECCC, 2023), and marine gas oil composition and properties are from Sørheim (2016). Oil composition and properties are shown in Table S1.

**Table S1.** Oil properties for Chayvo #6 Russian crude oil and marine gas oil

| Property | Chavyo #6 Russian Crude | Marine Gas Oil |
|---|---|---|
| API gravity | 38 | 35.16 |
| Density @ 15.6°C (g/cm$^3$) | 0.834 | 0.849 |
| Viscosity @ 20°C (cp) | 2.47 | 5.12 |
| Interfacial tension @ 15°C (dyne/cm) | 15.8 | 27 |
| Pour point (°C) | -4 | -36 |
| Fraction mono-aromatic hydrocarbons (MAHs) | 0.225 | 0.010 |
| Fraction 2-ring polycyclic aromatic hydrocarbons (PAHs) | 0.157 | 0.001 |
| Fraction 3-ring PAHs | 0.009 | 0.003 |
| Fraction non-aromatics: boiling point < 180°C | 0.013 | 0 |
| Fraction non-aromatics: boiling point: 180–264°C | 0.215 | 0.521 |
| Fraction non-aromatics: boiling point 265–380°C | 0.221 | 0.465 |
| Maximum mousse water content (%) | 64* | 0 |

*Assumed to be similar to MC252 oil, as analyzed by Belore et al. (2011)

**Figure S1 (next page).** Probability (%) of surface oil exposure ≥1 g/m2 (~1 µm; averaged over each ~0.75 km2 computational grid cell), based on a range of possible trajectories for a) release into the Chukchi Sea; b) October crude oil release into the Chukchi Sea; c) August marine gas oil release into the Chukchi Sea; d) October marine gas oil release into the Chukchi Sea; e) October crude oil release in the Bering Strait. Note map scale is the same for a, b and e and for c and d.

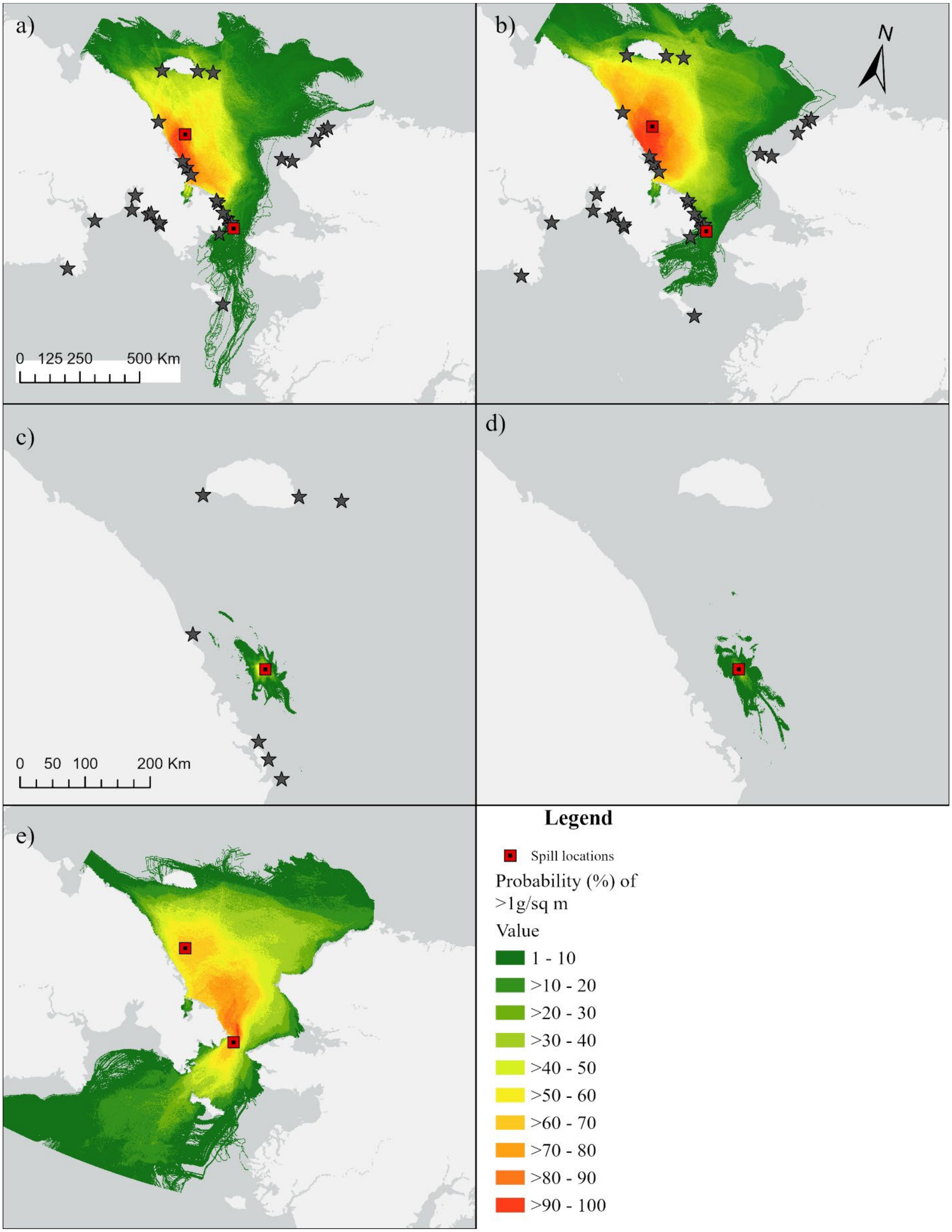
a)
0 125 250 500 Km
b)
N
c)
0 50 100 200 Km
d)
e)
Legend
Spill locations
Probability (%) of >1g/sq m
Value
1 - 10
>10 - 20
>20 - 30
>30 - 40
>40 - 50
>50 - 60
>60 - 70
>70 - 80
>80 - 90
>90 - 100

**References**


Belore, R. C., B. K. Trudel and J. Morrison. 2011. Weathering, emulsification, and chemical dispersibility of Mississippi Canyon 252 crude oil: Field and Laboratory studies. In: Proceedings of the 2011 International Oil Spill Conference. Abs. 247. DOI: 10.7901/2169-3358-2011-1-247

Dee, D. P., S. M. Uppala, A. J. Simmons, P. Berrisford, P. Poli, S. Kobayashi, U. Andrae, M. A. Balmaseda, G. Balsamo, P. Bauer, P. Bechtold, A. C. M. Beljaars, L. Van De Berg, J. Bidlot, N. Bormann, C. Delsol, R. Dragani, M. Fuentes, A. J. Geer, L. Haimberger, S. B. Healy, H. Hersbach, E. V. Hólm, L. Isaksen, P. Kållberg, M. Köhler, M. Matricardi, A. P. McNally, B. M. Monge-Sanz, J. -J. Morcrette, B. -K. Park, C. Peubey, P. De Rosnay, C. Tavolato, J. -N. Thépaut, and F. Vitart. 2011. The ERA-Interim reanalysis: configuration and performance of the data assimilation system. Quarterly Journal of the Royal Meteorological Society 137:553–597.

Environment and Climate Change Canada, 2023. Environment Canada Crude Oil and Petroleum Product Database. Environment Canada, Emergency Science and Technology Section. https://open.canada.ca/data/en/dataset/53c38f91-35c8-49a6-a437-b311703db8c5 Accessed 16 July 2024

Garcia H. E., T. P. Boyer, O. K. Baranova, R. A. Locarnini, A. V. Mishonov, A. Grodsky, C. R. Paver, K. W. Weathers, I. V. Smolyar, J. R. Reagan, D. Seidov, M. M. Zweng. 2019. World Ocean Atlas 2018: Product Documentation. A. Mishonov, Technical Editor.

Kullenberg, G., editor. 1982. Pollutant Transfer and Transport in the Sea, Volume I, CRC Press, Boca Raton, Florida, USA.

Okubo, A., 1971. Oceanic Diffusion Diagrams. Journal of Deep-Sea Research, 8:789–802.

Okubo, A. and R. V. Ozmidov, 1970. Empirical Dependence of the Coefficient of Horizontal Turbulent Diffusion in the Ocean on the Scale of the Phenomenon in Question. Atmospheric and Ocean Physics, 6(5):534–536.

Sørheim, K. R. 2016. Physical and Chemical Analyses of Crude and Refined Oils: Laboratory and Mesoscale Oil Weathering. OCS Study BOEM 2016-062. Prepared by SINTEF Materials and Chemistry and SEA Consulting Group for USDOI, Bureau of Ocean Energy Management, Alaska OCS Region, Anchorage, Alaska, USA. 106 pp. http://www.ntis.gov/products/publications.aspx Accessed 16 July 2024
